# Treatment of heart attack patients depends on history of cancer

**Published:** 2017

Treatment of heart attack patients depends on their history ofcancer, according to research published recently in EuropeanHeart Journal: Acute Cardiovascular Care. The study in morethan 35 000 heart attack patients found they were less likelyto receive recommended drugs and interventions and morelikely to die in hospital if they had cancer than if they did not.

‘It is well known that cancer patients may have an increased risk of cardiovascular disease as a result of their treatment’, said senior author Dr Dragana Radovanovic, head of the AMIS Plus Data Centre in Zurich, Switzerland. ‘However, on the other hand, little is known about the treatment and outcomes of cancer patients who have an acute myocardial infarction.’

This study investigated whether acute myocardial infarction patients with a history of cancer received the same guideline-recommended treatment and had the same in-hospital outcomes as those without cancer. The study included 35 249 patients enrolled in the Acute Myocardial Infarction in Switzerland (AMIS Plus) registry between 2002 and mid-2015. Of those, 1 981 (5.6%) had a history of cancer.

Propensity score matching was used to create two groups of 1 981 patients each – one with a cancer history and one without – that were matched for age, gender and cardiovascular risk factors. The researchers compared the proportions of patients in each group who received specific immediate drug therapies for acute myocardial infarction, and percutaneous coronary intervention (PCI) to open blocked arteries. They also compared the rates of in-hospital complications and death between the two groups.

The researchers found that cancer patients underwent PCI less frequently [odds ratio (OR) 0.76; 95% confidence interval (CI): 0.67–0.88) and received P2Y12 blockers (OR 0.82; 95% CI: 0.71–0.94) and statins (OR 0.87; 95% CI: 0.76–0.99) less frequently. In-hospital mortality rate was significantly higher in patients with cancer than those without (10.7 vs 7.6%; OR 1.45; 95% CI: 1.17–1.81).

Patients with a history of cancer were more likely to have complications while in hospital. They had 44% higher odds of cardiogenic shock, 47% higher chance of bleeding and 67% greater odds of developing heart failure than those with no history of cancer.

Dr Radovanovic said: ‘Patients with a history of cancer were less likely to receive evidence-based treatments for myocardial infarction. They were 24% less likely to undergo PCI, 18% less likely to receive P2Y12 antagonists and 13% less likely to receive statins. They also had more complications and were 45% more likely to die while in hospital.’

‘More research is needed to find out why cancer patients receive suboptimal treatment for myocardial infarction and have poorer outcomes’, continued Dr Radovanovic. ‘Possible reasons could be the type and stage of cancer, or severe co-morbidities. Some cancer patients may have a very limited life expectancy and refuse treatment for myocardial infarction’, she added.
